# Association Between Changes in Depressive Symptoms and Hazardous Drinking: Findings From the Korea Welfare Panel Study (2013–2018)

**DOI:** 10.3389/fpubh.2021.809043

**Published:** 2022-01-04

**Authors:** Sung Hoon Jeong, Doo Woong Lee, Soo Hyun Kang, Seung Hoon Kim, Eun-Cheol Park, Jaeyong Shin

**Affiliations:** ^1^Department of Public Health, Graduate School, Yonsei University, Seoul, South Korea; ^2^Institute of Health Services Research, Yonsei University, Seoul, South Korea; ^3^Department of Preventive Medicine, Yonsei University College of Medicine, Seoul, South Korea

**Keywords:** alcoholism, depression, hazardous drinking, Korea Welfare Panel Study, longitudinal data

## Abstract

**Objective:** This study aimed to evaluate the longitudinal association between changes in depressive symptoms and hazardous drinking among South Korean adults.

**Participants/Methods:** This study was conducted using a sample drawn from participants enrolled in the Korea Welfare Panel Study (KoWePS) from 2013 to 2018. Hazardous drinking was defined as a score of 11 points for men and seven points for women on the Korean version of the Alcohol Use Disorders Identification Test. Depressive symptoms were evaluated using an 11-item version of the Center for Epidemiologic Studies Depression Scale. A generalized estimating equation model was used to analyze the association between changes in depressive symptoms and hazardous drinking.

**Results:** Of the 12,878 people registered with KoWePS and without follow-up losses from 2013 to 2018, a total of 2,341 were included in this study, excluding those under the age of 19 and those with missing data. Persistent depressive symptoms (men, odds ratio [OR]: 2.20, 95% confidence interval [CI]: 1.48–3.21; women, OR: 2.64, 95% CI: 1.66–4.22) and the changes from non-depressive symptoms to depressive symptoms (men, adjusted OR: 2.18, 95% CI: 1.80–2.64; women, OR: 1.71, 95% CI: 1.27–2.29) increased the likelihood of hazardous drinking.

**Conclusions:** Persistent depressive symptoms and changes from non-depressive to depressive symptoms are associated with increased prevalence of hazardous drinking. This suggests that an evaluation of the underlying mental illness or emotion should be made when counseling for abstaining from alcohol for chronic drinkers or the general public.

## Introduction

Alcohol is the most commonly used psychoactive substance in the world; thus, health problems related to alcohol consumption are very important public health issues (1). According to the World Health Organization (WHO) (2), ~6% of all primary diseases can be attributed to harmful alcohol use (2). In addition to the recognized alcohol-related disorders, such as dependence or harmful use, there is growing interest in effects of hazardous drinking (HZD) of alcohol, which is defined as the level of drinking or drinking pattern that, if sustained, is likely to cause harm (2).

The WHO defines HZD as the amount or pattern of alcohol consumption that puts an individual at risk for unhealthy events and recognizes it as a distinct disability (3). HZD has been also defined as “a level of alcohol intake that is likely to cause harm if the current drinking habit continues” (4) or as “repetitive drinking patterns that can cause reasonably high harm” (5). In addition, HZD is defined as a level of alcohol consumption that poses a risk but does not meet the diagnostic criteria for alcohol use disorder (AUD) (6). These definitions clearly indicate that HZD can be harmful. Furthermore, it is associated with individual psychological and social problems (7, 8).

Depression is one of the most prevalent psychiatric disorders associated with alcohol consumption (9). A suggested mechanism behind this relationship is that people with depression tend to drink alcohol to cope with their depressive mood (“self-treatment”). Furthermore, compared to other psychoactive substances, alcohol is more commonly used as a form of coping because of its availability and acceptability in society (Self-medication Hypothesis) (10). However, the effects of alcohol on emotional/mental conditions such as depression are complex and remain controversial. Moderate alcohol consumption may relieve stress by temporarily alleviating negative emotional states in individuals (11), but frequent alcohol consumption may essentially lead to depression (12). In turn, people with depression are likely to increase alcohol consumption to relieve temporary emotional distress (13).

Over the past few years, the relationship between depression and alcohol consumption has been studied mainly through cross-sectional studies on the prevalence of AUD. The results of these studies suggest t that, in several cases, the correlation between depression and alcohol consumption is bidirectional (14). In other words, studies show that individuals with an alcohol consumption problem are at a higher risk of developing depression than those without an alcohol consumption problem, and people with depression have a relatively higher risk of developing AUD than those without depression (15–17). Despite this bidirectional relationship between depression and AUD, a prospective study in Denmark concluded that the causal role of AUD in depression was stronger than that of depression in AUD (18). However, most studies have focused on the changes in depression caused by alcohol consumption. Therefore, it is necessary to properly evaluate the influence of changes in depression (e.g., from depressed to non-depressed state) on alcohol consumption. Furthermore, most research has focused on AUD (19), despite the fact that HZD can also pose a great risk to an individual's health (3). Therefore, there is a need to investigate the relationship between changes in depression and HZD.

In addition, most previous studies examining the relationship between depression and alcohol consumption have been conducted using clinical groups such as individuals with an alcohol consumption problem or patients (20–23). Moreover, the studies involving non-clinical groups did not represent the general population, as their cohorts mostly included individuals from specific populations, such as workers (24) and students in middle school, high school, or university (25).

To the best of our knowledge, few epidemiological studies have investigated the relationship between HZD and changes in depression in the general Asian population. Therefore, this study aimed to investigate the association between changes in depressive symptoms and HZD in the general South Korean population using nationally representative longitudinal data.

## Methods

### Data and Population

The data used in this study were extracted from the Korean Welfare Panel Study (KoWePS). KoWePS is a nationally representative dataset of households in rural and urban areas, which was collated using layered multi-stage probability sampling. Individuals in selected households were interviewed face-to-face each year from January to February using a computer-aided personal interview technique. Since the commencement of the first KoWePS survey in 2006, a structured questionnaire has been administered annually to ~15,000–18,000 individuals in ~7,000 households, who are resampled annually to identify those who drop out of the survey. Details on the sample design, method, and dataset can be found at http://www.koweps.re.kr/. Since the data used in this study are publicly available, ethics approvals from the institutional review boards of the authors' affiliated institutions were not needed.

**Figure 1:**
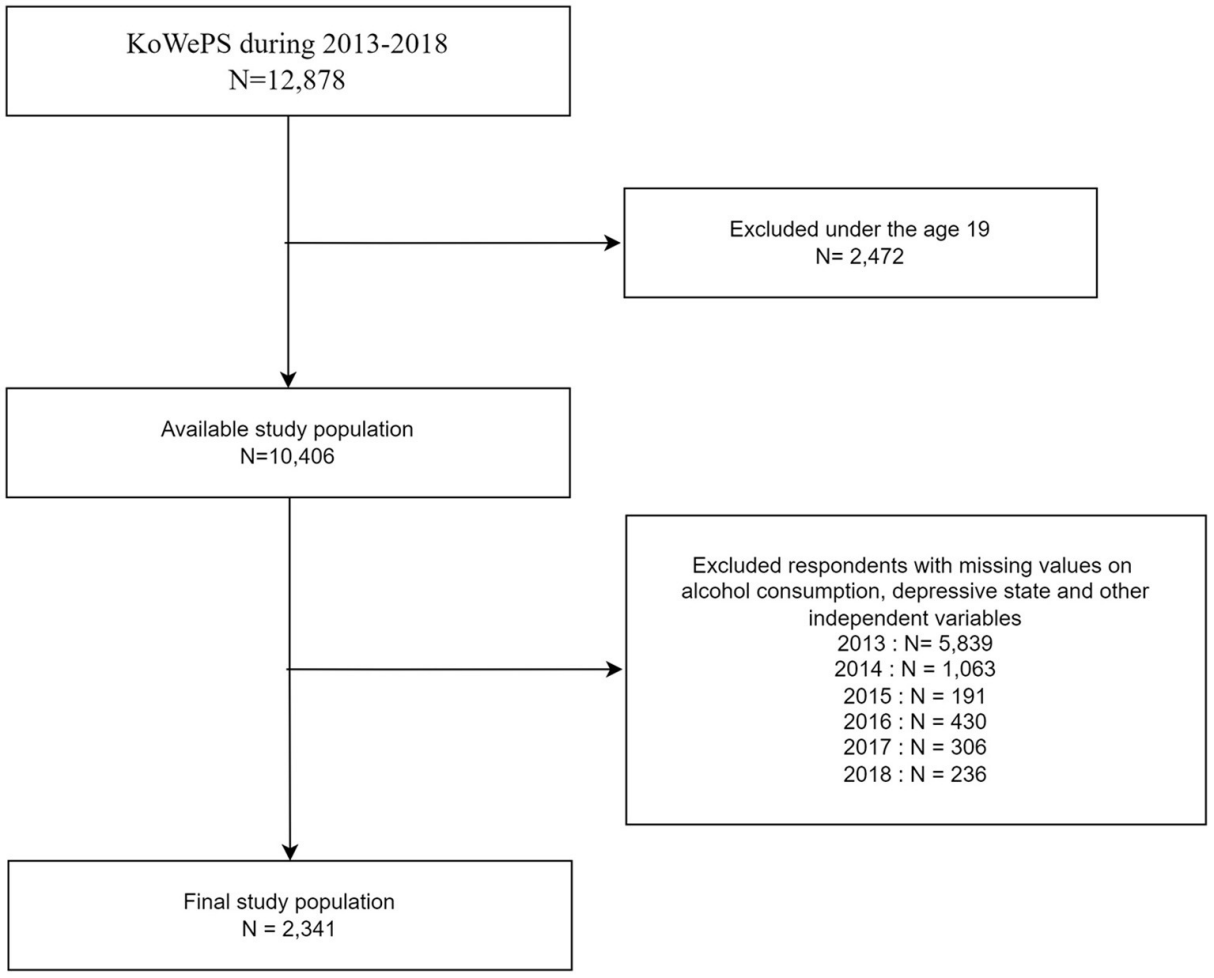
Flow diagram displaying the inclusion and exclusion of subjects.

This study was conducted using data collected over a duration of 6 years, from the 7th KoWePS survey cycle in 2013 to the 13 cycle in 2018. From the 2013 baseline data to 2018, of the 12,878 participants without of follow-up losses, participants under the age of 19 and those with missing data were excluded. After exclusion, the 2013 data set included a total of 2,341 individuals (1,783 males and 558 females) (Figure 1).

### Evaluation of Hazardous Drinking

In the KoWePS, a 10-item Alcohol Use Disorders Identification Test (AUDIT) scored on a 5-point Likert scale, was developed by the WHO to measure the level of harmful alcohol consumption (26). The total score ranges from 0 to 40 points, and high scores indicate high levels of harmful drinking. According to the WHO, the standardized AUDIT score for HZD is more than 8 points (27). However, a large standardization study of the Korean version of the AUDIT showed that 11 and 7 were the cut-off scores for HZD for men and women, respectively (22, 28), and the same reference points were used in this study.

### Evaluation of Depressive Symptoms

The main independent variable in this study was the change in depressive state, which was measured using the 11-item version of the Center for Epidemiologic Studies Depression Scale (CESD-11). The CESD-11 is a short version of the original 20-item version and is a well-validated self-report screening tool (29). The total score of the CESD-11 is calculated by adding the scores for all 11 questions and multiplying it by 20/11. A score of 16 or higher indicates the presence of depressive symptoms (30). To examine the year-to-year changes in depressive symptoms during the study period, we created four categories as follows: No → No (no depressive symptoms in the previous year and in the present year), No → Yes (no depressive symptoms in the previous year, but depressive symptoms in the present year), Yes → No (depressive symptoms in the previous year, but no depressive symptoms in the present year), and Yes → Yes (depressive symptoms in the previous year and in the present year).

### Covariates

Other demographic, socioeconomic, and health-related factors were included in the analysis. Sex, age, and region were the demographic variables. Education level, marriage status, and income level (quartile) were the socioeconomic variables. Smoking status and the absence or presence of chronic disease were the health-related factors.

### Statistical Analyses

We examined the distribution of the general characteristics of the study population at baseline (2013–2014). The chi-square tests were used to describe the general characteristics between individuals who had hazardous drinking or not at the 2013 baseline year. Taking into account the longitudinal nature of the data, we used a generalized estimation equation (GEE) model for repeated measures analysis and applied a lag function to determine the presence or absence of depression in the preceding and following years. A total of six waves (2013–2018) were used for the analysis, and since our study population was all traceable participants for 6 years, the measurement was repeated five times per person. We investigated the risk of HZD with changes in depressive symptoms for one consecutive year. The GEE model is an efficient and unbiased regression model used for the analysis of longitudinal or repeated measures in research designs with non-normal response variables (31). GEE analysis and calculations expressed as odds ratios (ORs) and 95% confidence intervals (CIs) were used to investigate the association between changes in depressive symptoms and HZD. Furthermore, we performed sensitivity analysis by changing the AUDIT score to 8 (26), which is the WHO HZD cut-off score, and 12 (32), which is the Korean alcohol use disorders cut-off score (for both men and women). Sex-stratified subgroup analyses were conducted to investigate the influence of age, educational level, marital status, employment status, and absence or presence of chronic diseases on the relationship between changes in depressive symptoms and HZD.

## Results

Table 1 presents the general characteristics of the study population at the first change timepoint (2013 to 2014). Of the 1,783 men included in this study, 642 (36.0%) were included in the HZD group, whereas 1,141 (64.0%) were not. The rate of change in depressive symptoms for men in the HZD group was 88.0% for the No → No group, 4.9% for the No → Yes group, 5.5% for the Yes → No group, and 1.6% for the Yes → Yes group. Of the 558 women included in this study, 151 (27.1%) were included in the HZD group, whereas 407 (72.9%) were not. The rate of change in depressive symptoms for women in the HZD group was 84.9% for the No → No group, 6.5% for the No → Yes group, 5.7% for the Yes → No group, and 2.9% for the Yes → Yes group.

**Table 1:** General characteristics of the study population at the first change time-point (2013 → 2014).

**Variables**	**Hazardous drinking**													
	**Total**		**Male**					**Total**		**Female**				
		**No (<11)**		**Yes (≥11)**			***P*-value**	**No (<7)**		**Yes (≥7)**				***P*-value**
	**N**	**%**	**N**	**%**	**N**	**%**		**N**	**%**	**N**	**%**	**N**	**%**	
Total	1,783	100.0	1,141	64.0	642	36.0		558	100.0	407	72.9	151	27.1	
Change of depression status (2013–2014)							0.0002							0.0045
No → No	1,569	88.0	1,029	90.2	540	84.1		474	84.9	356	87.5	118	78.1	
Yes → No	98	5.5	44	3.9	54	8.4		32	5.7	22	5.4	10	6.6	
No → Yes	87	4.9	53	4.6	34	5.3		36	6.5	23	5.7	13	8.6	
Yes → Yes	29	1.6	15	1.3	14	2.2		16	2.9	6	1.5	10	6.6	
Age							0.0018							0.2099
19–29	125	7.0	96	8.4	29	4.5		100	17.9	68	16.7	32	21.2	
30–39	418	23.4	274	24.0	144	22.4		160	28.7	113	27.8	47	31.1	
40–49	455	25.5	267	23.4	188	29.3		170	30.5	124	30.5	46	30.5	
50–59	374	21.0	230	20.2	144	22.4		70	12.5	53	13.0	17	11.3	
≥60	411	23.1	274	24.0	137	21.3		58	10.4	49	12.0	9	6.0	
Region							0.0561							0.0013
Metropolitan	781	43.8	519	45.5	262	40.8		284	50.9	224	55.0	60	39.7	
Rural	1,002	56.2	622	54.5	380	59.2		274	49.1	183	45.0	91	60.3	
Educational level							0.0009							0.3366
Middle school or less	392	22.0	238	20.9	154	24.0		105	18.8	82	20.1	23	15.2	
High school	643	36.1	387	33.9	256	39.9		219	39.2	154	37.8	65	43.0	
College or over	748	42.0	516	45.2	232	36.1		234	41.9	171	42.0	63	41.7	
Marriage status							0.6448							0.1512
Living w/o spouse	447	25.1	282	24.7	165	25.7		224	40.1	156	38.3	68	45.0	
Living w spouse	1,336	74.9	859	75.3	477	74.3		334	59.9	251	61.7	83	55.0	
Income							0.3592							0.4667
Low	256	14.4	152	13.3	104	16.2		63	11.3	50	12.3	13	8.6	
Lower middle	352	19.7	233	20.4	119	18.5		111	19.9	79	19.4	32	21.2	
Upper middle	525	29.4	336	29.4	189	29.4		186	33.3	139	34.2	47	31.1	
High	650	36.5	420	36.8	230	35.8		198	35.5	139	34.2	59	39.1	
Employment status							0.0317							0.0756
Permanent employee	720	40.4	479	42.0	241	37.5		147	26.3	106	26.0	41	27.2	
Temporary employee	345	19.3	217	19.0	128	19.9		164	29.4	116	28.5	48	31.8	
Employer or self- employed	454	25.5	267	23.4	187	29.1		39	7.0	23	5.7	16	10.6	
Unemployed or unpaid family worker	264	14.8	178	15.6	86	13.4		208	37.3	162	39.8	46	30.5	
Current smoking status							< .0001							< .0001
Smoker	885	49.6	505	44.3	380	59.2		34	6.1	13	3.2	21	13.9	
Non-smoker	898	50.4	636	55.7	262	40.8		524	93.9	394	96.8	130	86.1	
Chronic diseases							0.6281							0.0724
No	1,028	57.7	653	57.2	375	58.4		366	65.6	258	63.4	108	71.5	
Yes	755	42.3	488	42.8	267	41.6		192	34.4	149	36.6	43	28.5	

**Figure 2:**
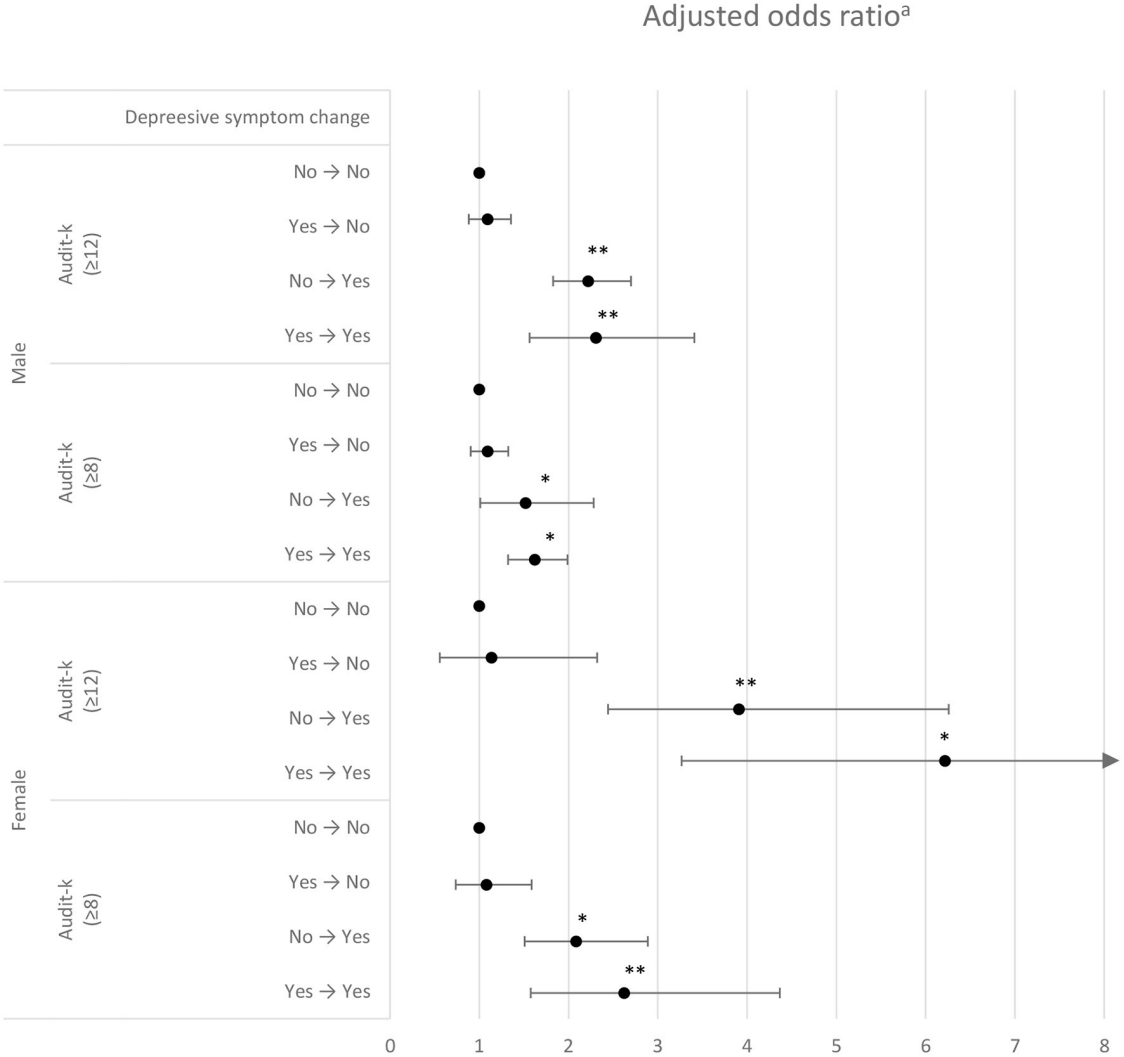
Sensitivity analysis using the generalized estimation equation for association between changes in depressive symptoms and hazardous drinking by different criteria for defining hazardous drinking.

Table 2 shows the GEE analysis examining the association between changes in depressive symptoms and HZD. Among men, the Yes → Yes group had the highest OR for HZD (OR = 2.20; 95% CI, 1.48–3.21). In addition, the No → Yes group had a higher OR for HZD than the No → No group (reference group) (OR = 2.18; 95% CI, 1.80–2.64). Among women, the Yes → Yes group also had the highest OR for HZD (OR = 2.64; 95% CI, 1.66–4.22), and the No → Yes group had a higher OR for HZD than the No → No group (OR = 1.71; 95% CI, 1.27–2.29). The results of the sensitivity analysis of the association between changes in depressive symptoms and HZD according to different definitions showed consistent results statistically (Figure 2).

**Table 2:** Results of the generalized estimating equation analysis of factors associated with Hazardous drinking.

**Variables**	**Hazardous drinking**							
	**Male (≥11)**				**Female (≥7)**			
	**OR**	**95% CI**				**OR**		**95% CI**
**Change of depression status**
No → No	1.00					1.00			
Yes → No	1.11	(0.91)	-	(1.36)		1.01	(0.73)	-	(1.41)
No → Yes	2.18	(1.80)	-	(2.64)		1.71	(1.27)	-	(2.29)
Yes → Yes	2.20	(1.48)	-	(3.21)		2.64	(1.66)	-	(4.22)
**Age**
19–29	1.00					1.00			
30–39	1.21	(0.89)	-	(1.63)		0.99	(0.68)	-	(1.44)
40–49	1.47	(1.07)	-	(2.01)		0.83	(0.55)	-	(1.26)
50–59	1.31	(0.93)	-	(1.83)		0.57	(0.35)	-	(0.92)
≥60	0.95	(0.66)	-	(1.35)		0.19	(0.10)	-	(0.38)
**Region**
Metropolitan	1.00					1.00			
Rural	1.02	(0.90)	-	(1.16)		1.20	(0.94)	-	(1.54)
**Educational level**
Middle school or less	1.00					1.00			
High school	0.93	(0.77)	-	(1.13)		0.89	(0.55)	-	(1.42)
College or over	0.72	(0.57)	-	(0.91)		0.57	(0.34)	-	(0.97)
**Marriage status**
Living w/o spouse	1.00					1.00			
Living w spouse	1.04	(0.89)	-	(1.22)		0.75	(0.55)	-	(1.01)
**Income**
Low	1.00					1.00			
Lower middle	1.16	(0.99)	-	(1.36)		1.38	(0.98)	-	(1.94)
Upper middle	1.22	(1.03)	-	(1.43)		1.18	(0.84)	-	(1.66)
High	1.40	(1.18)	-	(1.67)		1.36	(0.96)	-	(1.93)
**Employment status**
Permanent employee	1.05	(0.87)	-	(1.27)		1.19	(0.93)	-	(1.53)
Temporary employee	1.15	(0.95)	-	(1.38)		1.25	(0.98)	-	(1.59)
Employer or self- employed	1.25	(1.04)	-	(1.51)		1.60	(1.11)	-	(2.31)
Unemployed or unpaid family worker	1.00					1.00			
**Current smoking status**
Smoker	1.00					1.00			
Non-smoker	0.64	(0.57)	-	(0.72)		0.38	(0.26)	-	(0.57)
**Chronic diseases**
No	1.00					1.00			
Yes	0.98	(0.89)	-	(1.09)		0.97	(0.81)	-	(1.16)
**Year**
2014	1.00					1.00			
2015	1.01	(0.90)	-	(1.14)		1.42	(1.14)	-	(1.76)
2016	1.06	(0.94)	-	(1.19)		1.61	(1.29)	-	(2.01)
2017	0.91	(0.80)	-	(1.02)		1.47	(1.18)	-	(1.83)
2018	0.92	(0.82)	-	(1.05)		1.50	(1.19)	-	(1.89)

Table 3 shows the results of the stratified subgroup analysis of the association between changes in depressive symptoms and HZD according to marriage status, employment status, current smoking status and the absence or presence of chronic diseases by sex. In the subgroup analysis according to marital status, the OR for HZD was the highest among men and women with Yes → Yes depressive symptom changes who did not live with their spouse (OR = 2.19, 95% CI = 1.23–3.92 and OR = 1.79, 95% CI = 1.30–2.43, respectively). Regarding employment status, the OR for HZD was the highest among men who were permanent employees and women who were unemployed or unpaid family workers with Yes → Yes depressive symptom changes (OR = 2.29, 95% CI = 1.62–3.25 and OR = 1.95, 95% CI = 1.41–2.69, respectively). Regarding current smoking status, the OR for HZD was the highest among men who were smoking status and women who were non-smoking status with Yes → Yes depressive symptom changes (OR = 2.18, 95% CI = 1.33–3.57 and OR = 2.27, 95% CI = 1.31–3.94, respectively). For chronic diseases, the OR for HZD was the highest among men and women with chronic diseases who had Yes → Yes depressive symptom changes (OR = 2.68, 95 % CI = 1.75–4.10 and OR = 1.94, 95 % CI = 1.38–2.72, respectively).

**Table 3:** Subgroup analysis of the association between changes in depressive symptoms and hazardous drinking according to independent variables.

**Variables**	**Hazardous drinking**												
	**Change of depression status**												
	**No → No**			**Yes → No**			**No → Yes**				**Yes → Yes**		
	**OR**	**OR**	**95% CI**	**OR**	**95% CI**	**OR**	**95% CI**
**Men**
**Marriage status** ^**a**^
Living w/o spouse	1.00	1.14	(0.82)	-	(1.60)	2.17	(1.58)	-	(2.98)	2.19	(1.23)	-	(3.92)
Living w spouse	1.00	1.09	(0.84)	-	(1.40)	2.19	(1.72)	-	(2.79)	2.09	(1.21)	-	(3.62)
**Employment status** ^**b**^
Permanent employee	1.00	1.20	(0.83)	-	(1.72)	2.28	(0.79)	-	(6.59)	2.29	(1.62)	-	(3.25)
Temporary employee	1.00	1.31	(0.84)	-	(2.06)	2.17	(1.47)	-	(3.20)	1.93	(0.70)	-	(5.30)
Employer or self- employed	1.00	0.78	(0.48)	-	(1.26)	2.09	(1.38)	-	(3.18)	2.30	(1.39)	-	(3.79)
Unemployed or unpaid family worker	1.00	0.78	(0.48)	-	(1.26)	2.09	(1.38)	-	(3.18)	2.30	(1.39)	-	(3.79)
**Chronic diseases** ^**c**^
No	1.00	1.16	(0.89)	-	(1.51)	2.17	(1.67)	-	(2.82)	1.51	(0.74)	-	(3.09)
Yes	1.00	1.10	(0.81)	-	(1.49)	2.24	(1.67)	-	(2.99)	2.68	(1.75)	-	(4.10)
**Current smoking status** ^**d**^
Smoker	1.00	0.96	(0.75)		(1.22)	2.11	(1.68)		(2.65)	2.18	(1.33)		(3.57)
Non-smoker	1.00	1.18	(0.88)		(1.57)	2.10	(1.60)		(2.76)	1.87	(1.12)		(3.13)
Women
**Marriage status** ^**a**^
Living w/o spouse	1.00	0.96	(0.73)	-	(1.25)	1.47	(1.17)	-	(1.84)	1.79	(1.30)	-	(2.43)
Living w spouse	1.00	1.07	(0.80)	-	(1.42)	1.34	(1.02)	-	(1.75)	1.78	(1.15)	-	(2.74)
**Employment status** ^**b**^
Permanent employee	1.00	0.99	(0.65)	-	(1.52)	0.97	(0.65)	-	(1.47)	2.13	(0.93)	-	(4.85)
Temporary employee	1.00	0.97	(0.64)	-	(1.45)	1.31	(0.93)	-	(1.85)	1.42	(0.87)	-	(2.30)
Employer or self- employed	1.00	-	-	-	-	-	-	-	-	-	-	-	-
Unemployed or unpaid family worker	1.00	0.87	(0.64)	-	(1.20)	1.51	(1.14)	-	(1.98)	1.95	(1.41)	-	(2.69)
**Chronic diseases** ^**c**^
No	1.00	1.18	(0.92)	-	(1.52)	1.38	(1.09)	-	(1.75)	1.62	(1.11)	-	(2.35)
Yes	1.00	0.89	(0.63)	-	(1.26)	1.37	(1.03)	-	(1.82)	1.94	(1.38)	-	(2.72)
**Current smoking status** ^**d**^
Smoker	1.00	-	-	-	-	-	-	-	-	-	-	-	-
Non-smoker	1.00	1.17	(0.85)		(1.59)	1.57	(1.17)	-	(2.11)	2.27	(1.31)	-	(3.94)

a *Adjusted for sociodemographic and health-related factor (age, region, educational level, income, employment status, current smoking status, chronic diseases and year)*.

b *Adjusted for sociodemographic and health-related factor (age, region, educational level, marriage status, income, current smoking status, chronic diseases and year)*.

c *Adjusted for sociodemographic and health-related factor (age, region, educational level, marriage status, income, employment status, current smoking status and year)*.

d *Adjusted for sociodemographic and health-related factor (age, region, educational level, marriage status, income, employment status, chronic diseases and year)*.

## Discussion

In this study, we investigated the longitudinal association between changes in depressive symptoms and HZD in South Korean adults using national panel data collated repeatedly over 6 years. The main finding of this study was that when the cut-off score for HZD was set at 11 for men and seven for women, the results showed that participants with persistent depressive symptoms (Yes → Yes depressive symptom change) were more likely to engage in HZD than those who were not in a persistent depressed state. Furthermore, a sensitivity analysis performed after defining HZD according to various criteria showed that individuals with persistent depression tend to have higher risks for HZD than those without persistent depression. It should be noted that negative changes in depression status (No → Yes or Yes → Yes) are relevant when accompanied by high AUDIT scores (≥12). It can be interpreted that there is a close relationship between changes in depressive symptoms and HZD because the relationship between the two can be observed even when the standard for HZD is lowered or raised. Although the criteria for hazardous alcohol use applied in this study are different, our findings support those of the previous studies (33, 34). Furthermore, the results of this study also support those of some previous studies that indicate that the level of alcohol consumption can be predicted using previous depression (35). In addition, the results of this study are unique because we investigated the association between HZD and depression with a focus on changes in depressive symptoms, using longitudinal data from a large sample that represented the general population of South Korea.

As observed in the results of this study, the mechanisms by which changes in depressive states lead to HZD can be explained in two ways. First, the self-medication hypothesis (36), which suggests that people who are generally depressed drink alcohol as a form of self-prescription to treat their depression (16). The self-medication hypothesis implies that alcohol abuse is an action that is unavoidably performed by a depressed person to control internal emotions and adapt to reality. The problem worsens through repeated ingestion of higher alcohol amounts to avoid feelings of loneliness and depression (36). Second, the tension reduction hypothesis (37), which suggests that people drink alcohol to relieve negative emotional states, such as anxiety, depression, and sadness, or to improve low self-esteem (33). In fact, alcohol has a pharmacological effect in the reduction of stress-induced tension or psychological distress. Owing to the nature of alcohol, people under tension or who experience anxiety, reinforce their drinking behavior in stressful situations (38). Simply put, people drink alcohol to relieve psychological distress symptoms, such as anxiety and depression, in order to cope with stressful situations. Based on our findings, we believe that this phenomenon is strengthened when depression persists.

The results of a sub-analysis in this study confirmed that Persistent depression has been shown to increase HZD among people who do not live with their spouse. Previous studies have shown that healthy marital status not only has a positive effect on treatment and rehabilitation but also alleviates alcoholism through social support (39). Some previous studies have shown that social support through healthy social activities can prevent alcoholism (40). Persistent depression increased HZD in permanently hired men employees. According to previous studies, Korean permanently workers work longer than Western workers (41). It was found that such long working hours cause job stress and lead to depression in many cases (42), and they choose alcohol to relieve stress (41). In addition, persistent depression in men smokers increased HZD. Compared to non-smokers, smokers were found to have a higher life-long major depressive episode (43). Furthermore, the higher the amount of smoking, the higher the probability of alcohol abuse or dependence on alcohol (43). As a result, persistent depression coexisted with smoking, suggesting an increase in HZD due to a synergistic effect. HZD has been found to be the highest in adults with chronic conditions and persistent depression. Perhaps, the physical pain experienced by patients with a chronic illness may serve as a motive for drinking. Furthermore, alcoholic drinks are used as a means to deal with negative emotions, such as persistent depression (14).

It is common to find individuals with HZD problems in primary care clinics. Therefore, recognition of HZD is widely regarded as a cost-effective way for general practitioners and other healthcare professionals to identify, evaluate, and advise patients in the preliminary stage of alcoholism disorder (44). Previous research suggests that people with AUD have a hard time returning to “controlled” drinking, whereas those who engage in HZD can achieve moderate alcohol intake goals (45). Therefore, appropriate preventive management measures for depression should be taken to prevent HZD. Furthermore, it is believed that these preventive measures could play a role in inhibiting the transition from HZD to AUD (46).

The main aim of this study was to evaluate the longitudinal association between changes in depressive symptoms and HZD in South Korean adults. Several cross-sectional studies have been conducted to evaluate the relationship between depression and AUD; however, causality and changes in variables over time were not evaluated in these studies (16). Nevertheless, a major strength of our research is that it focused on HZD, which is considered a predecessor to AUD. In addition, we used various alcohol intake criteria and conducted sensitivity analyses of variability in alcohol intake levels to confirm the association between persistent depressive symptoms and alcohol intake, thereby ensuring that the correlation revealed in the results is strong.

This study has some limitations. First, although the KoWePS data have been statistically verified to be nationally representative, the loss of some study samples due to the exclusion criteria of the present study may have reduced the external validity. Furthermore, selection bias may exist in this study due to the high exclusion rate. Second, as this study was based on longitudinal data observed repeatedly at different levels over a set period, we were unable to determine the perfect causal relationship between depressive changes and HZD; there is also the possibility of an inverse causal relationship. Third, it was difficult to devise a standard definition for HZD due to the various available definitions and criteria for hazardous alcohol consumption. However, based on the findings of previous studies, we performed a sensitivity analysis through various classifications using AUDIT and found a consistent association between changes in depressive state and HZD. Fourth, the use of self-reported responses means that measurement issues, such as reporting bias and recall bias, may have confounded the findings. Finally, there may be other issues related to the use of unobserved and uncontrolled confounding variables.

In this study, we investigated the association between changes in depressive symptoms and HZD in the general population. We found that persistent depressive symptoms and changes from non-depressive to depressive symptoms are associated with increased prevalence of HZD. These findings demonstrate that the evaluation of underlying mental illnesses or emotions should be conducted during abstinence counseling for chronic alcohol drinkers. Furthermore, abstinence education programs should be integrated into the treatment of depression symptoms.

## Data Availability Statement

The datasets presented in this study can be found in online repositories. The names of the repository/repositories and accession number(s) can be found below: All the KoWePS data used in this study are available to the public and can be seen in the KoWePS official website (https://www.koweps.re.kr:442/eng/main.do).

## Ethics Statement

The studies involving human participants were reviewed and approved by Institutional Review Board of Korea Institute for Health and Social Affairs (No. 2021–002). The patients/participants provided their written informed consent to participate in this study.

## Author Contributions

SJ, JS, and DL conceived and designed the study. SJ, SKa, and DL conducted the formal analysis and methodology. SJ wrote the initial drafts. SKi and E-CP helped draft the manuscript. JS is the corresponding author of this work and supervised the entire study and the manuscript. All authors have read and approved the final manuscript.

## Funding

This study was supported by a faculty research grant of Yonsei University College of Medicine for (6-2021-32-0040).

## Conflict of Interest

The authors declare that the research was conducted in the absence of any commercial or financial relationships that could be construed as a potential conflict of interest.

## Publisher's Note

All claims expressed in this article are solely those of the authors and do not necessarily represent those of their affiliated organizations, or those of the publisher, the editors and the reviewers. Any product that may be evaluated in this article, or claim that may be made by its manufacturer, is not guaranteed or endorsed by the publisher.
